# Evaluation of bactericidal effects of silver hydrosol nanotherapeutics against *Enterococcus faecium* 1449 drug resistant biofilms

**DOI:** 10.3389/fcimb.2022.1095156

**Published:** 2023-01-11

**Authors:** Alya Limayem, Mausam Mehta, Natalie Kondos, Divya Kaushal, Farhat Binte Azam, Sriram Chellappan, Nan Qin, Qingyu Zhou

**Affiliations:** ^1^ Department of Biology, College of Arts & Sciences, University of North Florida, Jacksonville, FL, United States; ^2^ Department of Pharmaceutical Sciences, Graduate Program, Taneja College of Pharmacy, University of South Florida, Tampa, FL, United States; ^3^ Morsani College of Medicine, University of South Florida, Tampa, FL, United States; ^4^ Department of Computer Science & Engineering, College of Engineering, University of South Florida, Tampa, FL, United States; ^5^ Department of R&D and Analytical Services, Natural Immunogenics Corporation, Sarasota, FL, United States

**Keywords:** multi-drug resistance, *E. faecium*, bioactive silver hydrosol nanoparticles, bactericidal treatment, cytotoxicity, artificial intelligence, computer vision

## Abstract

**Introduction:**

Silver (Ag) nanoparticles (NPs) are well documented for their broad-spectrum bactericidal effects. This study aimed to test the effect of bioactive Ag-hydrosol NPs on drug-resistant *E. faecium* 1449 strain and explore the use of artificial intelligence (AI) for automated detection of the bacteria.

**Methods:**

The formation of *E. faecium* 1449 biofilms in the absence and presence of Ag-hydrosol NPs at different concentrations ranging from 12.4 mg/L to 123 mg/L was evaluated using a 3-dimentional culture system. The biofilm reduction was evaluated using the confocal microscopy in addition to the Transmission Electronic Microscopy (TEM) visualization and spectrofluorimetric quantification using a Biotek Synergy Neo2 microplate reader. The cytotoxicity of the NPs was evaluated in human nasal epithelial cells using the MTT assay. The AI technique based on Fast Regional Convolutional Neural Network architecture was used for the automated detection of the bacteria.

**Results:**

Treatment with Ag-hydrosol NPs at concentrations ranging from 12.4 mg/L to 123 mg/L resulted in 78.09% to 95.20% of biofilm reduction. No statistically significant difference in biofilm reduction was found among different batches of Ag-hydrosol NPs. Quantitative concentration-response relationship analysis indicated that Ag-hydrosol NPs exhibited a relative high anti-biofilm activity and low cytotoxicity with an average EC50 and TC50 values of 0.0333 and 6.55 mg/L, respectively, yielding an average therapeutic index value of 197. The AI-assisted TEM image analysis allowed automated detection of *E. faecium* 1449 with 97% ~ 99% accuracy.

**Discussion:**

Conclusively, the bioactive Ag-hydrosol NP is a promising nanotherapeutic agent against drug-resistant pathogens. The AI-assisted TEM image analysis was developed with the potential to assess its treatment effect.

## 1 Introduction

During the last decade microbial infections from drug resistant bacteria and viruses have known an unprecedented spread and transmissibility, requiring urgent actions. Complicating these concerns are the existing bactericides, which are becoming progressively weak due to the increased drug resistance and continuous mutations. As a result, according to the CDC report [https://www.cdc.gov/drugresistance/biggest-threats.html], each year the United States experiences more than 2.8 million antibiotic resistant infections with over 35,000 fatalities. Nosocomial infections from multidrug resistant (MDR) *Enterococcus* alone comprise a serious threat to public health, registering an estimate of 54,500 hospitalizations and 5,400 deaths annually [https://www.cdc.gov/drugresistance/biggest-threats.html]. MDR bacteria like *Enterococci* have allowed isolates to survive multiple generations of anti-gram-positive therapeutics (Miller et al., 2014), requiring novel nanotherapeutics including metal oxides such as the chitosan delivering zinc oxide nanocomposites (CZNPs) (Limayem et al., 2020) or silver (Ag) hydrosol (Sim et al., 2018). Vancomycin-resistant *E. faecium* (VRE*)* are increasingly becoming a threat as a nosocomial infection (Johnston and Jaykus, 2004; Puchter et al., 2018). This species has become more serious as it is the most common species in its genus appearing in agricultural products, thus allowing the general population to be susceptible to severe infections (Johnston and Jaykus, 2004). As populations in countries like the United States become older, more vulnerable subgroups will be at risk of *E. faecium* in addition to the immunocompromised population as MDR spectrum in bacteria increases (Limayem et al., 2015).

Cases of MDR in *E. faecium* isolates started to increase between 1978 and 1998 (Huycke et al., 1998) and have been a legitimate threat worldwide for the past three decades (Arias and Murray, 2012; Georges et al., 2022; van Dijk et al., 2022; Coombs et al., 2022). As a major pathogen responsible for nosocomial infections, *E. faecium* isolates have often been found in surgical wound and urinary tract infections (Huang et al., 2021; Strobel et al., 2022). Epidemiological investigations have found that VRE caused hospital outbreaks in various occasions (Szakacs et al., 2014; Sivertsen et al., 2016; Hammerum et al., 2019; Abdullah et al., 2022), while *E. faecium* with dual resistance to linezolid and vancomycin was involved in the *E. faecium* outbreak in Reunion Island in 2019 (Kamus et al., 2022). In terms of specific MDR, antibiotics such as cephalosporins, macrolides and semi-synthetic penicillin-like beta-lactams demonstrate prominent levels of resistance (Guirao et al., 2009; Kristich et al., 2014; Georges et al., 2022). A previously successfully treatment, quinupristin-dalfopristin, has slowly reduced efficacy as *E. faecium* developed resistance towards that specific treatment (Lopez et al., 2010). Aminoglycosides are another class of antibiotics that *E. faecium* has demonstrated prominent levels of resistance (Troscianczyk et al., 2021). As *E. faecium* gained more MDR, less traditional antibiotic agents have demonstrated inhibitory or bactericidal activity. Compared to its fellow species *E. faecalis*, *E. faecium* has formed the bulk of most VRE infections (Ayobami et al., 2020; Penven et al., 2022). The phenotypic antimicrobial susceptibility testing results received at the French National Reference center revealed that none of the 34 *E. faecalis* clinical isolates was resistant to vancomycin, ampicillin, or teicoplanin, whereas majority of the *E. faecium* clinical isolates exhibited resistance to vancomycin (85.5%), ampicillin (99.3%), or teicoplanin (73.9%) (Penven et al., 2022). It is believed that intrinsic processes help *E. faecium* acquire MDR, such as mutations that promote resistance against tigecycline that probably remains the last effective antibiotic against MDR *E. faecium* (Cattoir and Giard, 2014), since mutations of the *rpsJ* gene encoding the 30S ribosome protein S10 could reduce tigecycline susceptibility (Cattoir et al., 2015). To this aim, novel type of drugs are of utmost need.

Silver (Ag) nanocomposites hold promises as bactericidal agents against the emerging MDR bacteria over conventional antibiotics and have been a potential antibiotic agent towards gram-positive and gram-negative bacteria including *E. faecium* and *Escherichia coli* (Gong et al., 2007; Rai et al., 2009; Khan et al., 2020).. They have also exhibited a strong bactericidal activity against the gram-negative bacteria, such as *Pseudomonas aeruginosa* and *Klebsiella pneumonia*, as well as the gram-positive bacteria, such as *Staphylococcus aureus* (Khan et al., 2011; Guzman et al., 2012; Rubio-Canalejas et al., 2022; Kabeerdass et al., 2022). Whether the bacterial species are gram-positive or gram-negative, Ag nanoparticles (NPs) manage to accumulate inside of the bacterial membrane and navigate across the peptidoglycan to cause cell damage (Sondi and Salopek-Sondi, 2004). Ag NPs can form ‘pits’ on the surface of the cellular membrane and produce free radicals to damage the cellular membrane and inactivate respiratory enzymes within the cell, leading to cell death (Prabhu and Poulose, 2012). Furthermore, the interaction of Ag NPs with bacterial species is dependent on the size and shape of the nanoparticles (Panacek et al., 2006; Pal et al., 2007). It was reported that the smaller the dimensions of the Ag nanoparticles are, the higher the bactericidal effect and potency (Panacek et al., 2006).

As with any drug or consumed substance, it is possible that if the dosage of Ag NPs consumed is beyond the safe threshold toxicity, it would generate a few side effects, requiring the determination of the reference dose and the therapeutic index (TI) before use (Gong et al., 2007). The TI of each type of Ag NPs can be estimated based on the ratio of the median lethal dose (LD_50_) to the median effective dose (ED_50_) (Mehta et al., 2019). The addition of a capping agent or a similar adjuvant, like polyethylene glycol (PEG), reduces cell surface charge and in increase the safe threshold of Ag NPs (Tortella et al., 2020). Ag NPs hold promises with their strong bactericidal potential against the emerging MDRs (Geilich et al., 2015). The particle size and surface properties of Ag NPs can be tailored through various methods such as spark discharging (Abd El-Aal et al., 2018), electrochemical reduction (Zhang et al., 2002), laser ablation (Boutinguiza et al., 2015), cryochemical synthesis (Vernaya et al., 2017) and green synthesis (Wypij et al., 2021).

The overall objective of this study was to determine the efficacy of bioactive Ag-hydrosol NPs received from Natural Immunogenics Corporation (NIC) on drug resistant *E. faecium* 1449. The novelty of this study, however, are two-fold: (1) the use of a 3-dimentional (3-D) culture system for MDR biofilm growth to mimic the extracellular collagen matrix within the *in vivo* conditions when exposed to Ag-hydrosol NPs; (2) the use of Automated Intelligence (AI) paralleled with the spectrofluorimetric quantification for a rapid detection of the biofilm shrinkage. The bioactive Ag-hydrosol NPs tested in this study owes its uniqueness to the refinement of its colloidal texture and unmatched size as small as 0.8 nm, making it easily absorbable by the host cell. The effectiveness of Ag-hydrosol NPs on *E. faecium 1449* biofilm reduction evaluated using a Biotek Synergy Neo2 microplate reader was the first of its kind to validate the biofilm shrinkage to a substantial level as high as 95.2%. Furthermore, the high TI value of 197 indicates a low risk of toxicity within the therapeutic dose range when correlated to a relatively low concentration of the treatment at as low as 12.4 mg/L.

## 2 Materials and methods

### 2.1 Materials and cell culture

The mutating Clonal Complex 17 MDR *E. faecium* 1449 strain was obtained as previously described (Limayem et al., 2015; Mehta et al., 2019). Ag-hydrosol NPs prepared at different concentrations were provided by NIC as follows: SHFa-1 (12.4 mg/L), SHFa-2 (12.4 mg/L), SHFa-3 (12.4 mg/L), SHFb-1 (26.5 mg/L), SHFb-2 (26.8 mg/L), SHFb-3 (27.0 mg/L), SHFc-1 (123.0 mg/mL).

Human nasal epithelial cells (Catalog number: C-12620) purchased from PromoCell GmbH (Heidelberg, Germany) were cultivated in the ready-to-use airway epithelial cell growth medium according to the manufacturer’s protocol (Catalog number: C-21060. PromoCell GmbH, Heidelberg, Germany).

### 2.2 Quantitative assessment of anti-biofilm activity of Ag-Hydrosol NPs using a microtiter plate assay

The anti-biofilm activity of Ag-hydrosol NPs was evaluated using a microtiter plate assay described elsewhere (O'Toole, 2011). Briefly, an aliquot of 50 μL of *E. faecium* 1449 culture diluted in 2 × fresh medium was added to each well of a 96-well plate followed by the addition of 50 μL of two different batches of Ag-Hydrosol NPs (SHFa-2 and SHFb-1) at various concentrations (0.0097 ~ 13.25 mg/L) and incubation of the microtiter plate at 37°C overnight. After the incubation, the microtiter plate was gently washing and biofilms were stained with 0.1% crystal violet solution. Optical density of each well was measured at 550 nm using the Biotek Synergy Neo2 hybrid multimode reader (Agilent Technologies. Santa Clara, CA, USA). The biofilm formation was expressed as a percentage of vehicle control *E. faecium* 1449 biofilms. Concentrations of Ag NPs required for 50% biofilm reduction (i.e., EC50) as compared with the vehicle control were calculated by log-linear fitting of the concentration–response curve of crystal violet staining as a function of Ag NP concentration obtained from experiments performed in triplicates using the GraphPad Prism 8.0 program (GraphPad Software, Inc. La Jolla, CA).

### 2.3 Evaluation of cytotoxic effect of Ag-Hydrosol NPs on human nasal epithelial cells by MTT assay

The cytotoxicity of Ag-hydrosol NPs in human nasal epithelial cells was determined using the MTT (3-(4,5-dimethylthiazol-2-yl)-2,5-diphenyltetrazolium bromide) assay described elsewhere with minor modifications (Zhou et al., 2017). Briefly, human nasal epithelial cells were seeded in 96-well plates at a density of 2×10E4 cells/100 μl/well and allowed to attach overnight. After the overnight incubation, cells were treated with two different batches of Ag-hydrosol NPs (SHFa-2 and SHFb-1) at different concentrations ranging from 0.388 to 13.3 mg/L for 24 hours. At the end of the treatment period, 10 μL of approximately 12 mM of MTT (Thermofisher Scientific, Waltham, MA) stock solution was added to each well and incubated for 2 hours. Following the incubation, the medium was removed from each well, to which 100 μL of DMSO was added to dissolve the formazan crystals. Optical densities were measured at 570 nm using the Biotek Synergy Neo2 hybrid multimode reader. The growth of treated cells was expressed as a percentage of vehicle control cultures. Concentrations of individual drugs required for 50% inhibition of cell growth (i.e., TC50) as compared with the control cells were calculated by log-linear fitting of the concentration-cell viability curve obtained from experiments performed in triplicates using the GraphPad Prism 8.0 program (GraphPad Software, Inc. La Jolla, CA) (Zhou et al., 2017).

### 2.4 Assessment of Ag-Hydrosol NP treatment effect on the constitution of *E. faecium* biofilm population and formation of extracellular polymeric substances using fluorescence confocal microscopy

The inert polystyrene Alvatex strata 3D scaffold inserts (ReproCELL Europe Ltd., Glasgow, UK) that mimic the structural *in vivo* conditions were utilized to grow *E. faecium* 1449 biofilms as previously described (Limayem et al., 2020). *E. faecium* 1449 biofilm matrices composed of extracellular polymeric substances (EPS) were treated with Ag NPs at various concentrations for 24 hours. Control and treated biofilms were stained before observed through the Olympus FV1000 confocal laser scanning microscopy (Olympus Co., Tokyo, Japan). To examine the constitution of *E. faecium* biofilm population, biofilms were stained using LIVE/DEAD Viability Assay Kit according to the manufacturer’s protocol (Thermo Fisher Scientific, Waltham, MA). The stained biofilms were then individually imaged with the confocal laser scanning microscope. To assess the EPS formation, *E. faecium* 1449 biofilms were stained with a combination of fluorescein isothiocyanate isomer I (FITC) and calcofluor white M2R (CFW) dyes for the visualization of protein (green fluorescence signals) and β-polysaccharide components of EPS (blue fluorescence signals) using confocal microscopy as previously described (Limayem et al., 2020).

### 2.5 Quantification of percent reduction of *E. faecium* cultured in 3D scaffolds

To quantify the biofilm reduction upon treatment, the remaining biofilms stained with the LIVE/DEAD Viability Assay Kit were placed in a 50 mL tube containing 10 mL of sterile phosphate-buffered saline (PBS) and sonicated at full power for 20 minutes. Upon completion, 200 µL of the resulting biofilm suspension was transferred to each well of a 96-microtiter plate. The Biotek Synergy Neo2 microplate reader (Agilent Technologies. Santa Clara, CA, USA) was then utilized to read the wells at the excitation/emission frequencies of 470/510 nm wavelength for live cells and 535/617 nm wavelength for dead cells. The resultant fluorescence values from the microplate reader for each treatment were compared with the positive (vehicle treatment) and negative (blank) controls. The percent biofilm reduction was calculated as


$$\%biofilm\;reduction=100\times \frac{raio\;of\;viable\;bacteria-ratio\;of\;dead\;bacteria}{\frac{1}{2}\times \left(raio\;of\;viable\;bacteria+ratio\;of\;dead\;bacteria\right)}$$

where ratios of viable and dead bacterial cells are defined as $\frac{abs_{treatment}-abs_{negative\;control}}{abs_{positive\;control}-abs_{negative\;control}}$ determined at an excitation/emission of 470/510 nm and 535/617 nm, respectively (Limayem et al., 2020).

### 2.6 Transmission electron microscopy and dynamic light scattering

The transmission electron microscopy (TEM) images of the Ag-hydrosol NPs were captured using a Phillips CM100 TEM operated at 100 kV. The TEM samples were prepared using the formvar-carbon coated 300 mesh copper TEM grid (Part# 71150, Electron Microscopy Services). A 1.0 μL drop was deposited onto the grid and allowed dry in a desiccator. The TEM images were analyzed by ImageJ (https://imagej.nih.gov/ij/index.html) to detect the particle outlines automatically. The particle diameters were calculated from the area within the outline assuming circular shapes.

The MER *E. faecium 1449* bacteria treated with Ag-hydrosol NPs were visualized using a JEOL JEM 1400 electron microscope with Gaton camera. The *E. faecium 1449* bacteria were treated with various batches of Ag-hydrosol NPs for 3 hours. Following the treatment, the bacteria were fixed in 2% paraformaldehyde and adsorbed to formvar coated TEM 200 mesh copper grids and settled for 2 hours under air-tight cover before the dry sample was imaged under the TEM.

Dynamic light scattering (DLS) was performed using the Malvern Zetasizer Ultra (Malvern Panalytical Ltd., Malvern, UK). The samples were used as is. A 10-second delay was placed prior to each successive measurement.

### 2.7 Statistical analysis

Statistical analysis was carried out using IBM SPSS version 27. Data are presented as the mean ± standard deviation (SD) unless otherwise indicated. Results of biofilm reduction and human nasal epithelial cell viability percentages were calculated from the observations of three independent experiments, and error bars represent the SD from the mean. A one-way ANOVA was performed to compare the difference in the means of percent reduction of biofilms of 3D *E. faecium* 1449 cultures among the different treatment groups.

### 2.8 Artificial intelligence (computer vision) techniques applied to TEM imaging for future automated evaluation

The goal of the artificial intelligence (AI) module in this cycle was to detect and localize the pili of *E. faecium* 1449 in the TEM images. We focused on both treated and untreated *E. faecium* 1449. Our data set for this evaluation included 28 images. Out of which 25 images were used for training and validation, while the remaining 3 images were used for testing alone. The basic architecture used for this study herein was Fast Regional Convolutional Neural Networks (R-CNNs) and was summarized in different steps as follows (Girshick and Fast, 2015):

a. The feature maps were extracted from the original images. They essentially represented the key attributes of an image in matrix form. For this problem, the standard ResNet 50 Deep neural network architecture were used.
b. Subsequently, the training images were hand annotated to carefully emplace bounding boxes on the foreground object of interest, which contains the pili in this case.
c. In the next step, the feature maps from the first step was process with the manually emplaced bounding boxes *via* a simpler convolutional neural network to identify features that represent the foreground object of interest (again, the pili of *E. faecium* 1449).
d. Steps a. and b. were trials and methods with various parameters called hyper-parameters that govern how the neural network works. Once validated with sufficient accuracy, the neural network architectures were finalized.
e. At runtime, the following steps were executed for an input image:
i. the ResNet 50 CNN identified the feature maps;
ii. a number of random bounding boxes of varying sizes were automatically emplaced in the input image that encompass the whole image;
iii. the extracted features from Step a. were processed with features derived from each emplaced box *via* the simpler which ultimately yielded to classification of each emplaced box into foreground and background, and also the emplaced boxes were tightened.

## 3 Results

### 3.1 Ag-Hydrosol NP characterization

**Figure 1:**
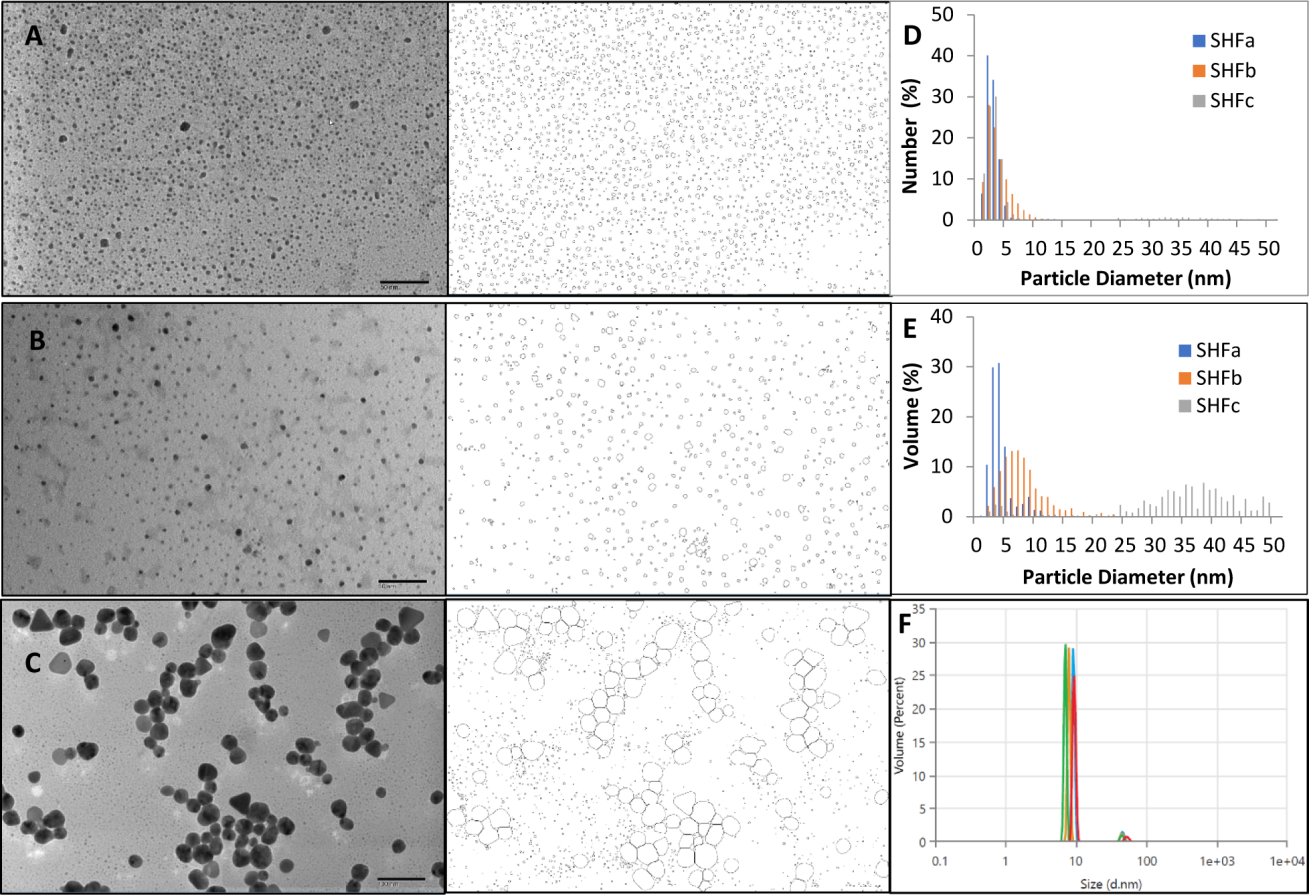
The representative TEM images and particle outlines of **(A)** SHFa, **(B)** SHFb and **(C)** SHFc. The nanoparticle size number distribution of the three products is shown in **(D)** and the volume distribution in **(E)** DLS particle volume distribution of SHFb is shown in **(F)**.

**Figures 1A–C** show representative TEM images of the three products of Ag-hydrosol NPs. The nanoparticles of SHFa and SHFb are mostly circular with majority of NPs fall under sub-10 nanometers. SHFc contains NPs with two groups of sizes, one group being distinctively larger than the other group. One can see that in the number distribution histogram (**Figure 1D**), majority of particles of all three samples are less than 10 nm. However, in the volume distribution graph (**Figure 1E**), majority of the NP volume of SHFc is attributed to the particle with sizes between 25 nm to 50 nm, meaning that most of the silver content is in the larger particles, despite the numerous sub-10 nanometer particles that are dominant in quantity. In contrast, both the number and volume distribution of SHFa and SHFc are in the sub-10 nm range, indicating highly uniformed and narrow particle size distribution. The volume distribution of SHFb measured by DLS is also presented in **Figure 1F**. The DLS volume distribution contains two peaks. Peak one accounts for 95% of total particle volume with mean particle size of 8.4 ± 1.1 nm while peak two accounts for 5% with mean particle size of 47.2 ± 3.8 nm. DLS measures the hydrodynamic diameter of particles whereas TEM measures the core particle size. The hydrodynamic diameter is typically slightly larger than the core size (Pabisch et al., 2012). Taken together, the DLS result is considerably in agreement with the TEM result.

### 3.2 Estimation of therapeutic index based on quantitative evaluation of anti-biofilm activity and cytotoxicity of Ag-Hydrosol NPs

**Figure 2:**
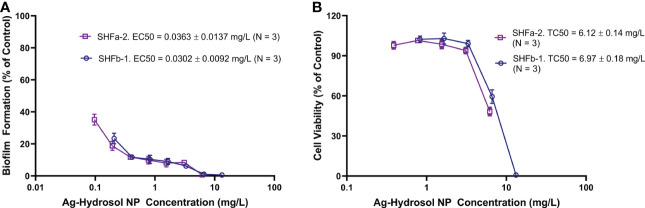
Quantitative evaluation of the anti-biofilm and cytotoxicity effects of Ag-hydrosol NPs. **(A)** Composite dose-effect curves for the effect of Ag-hydrosol NPs on *E faecium 1449* biofilm formation determined using the microtiter plate assay with crystal violet staining. **(B)** Composite dose-effect curves for the *in vitro* cytotoxic effect of Ag-hydrosol NPs on human nasal epithelial cells determined by the MTT assay. Error bars represent the standard deviation (SD) of three independent experiments (N = 3).

To characterize the relationship between the Ag-hydrosol NP concentration and its anti-biofilm activity and cytotoxicity, *E. faecium* 1449 bacteria and human nasal epithelial cells were treated with SHFa-2 and SHFb-1 Ag-hydrosol NPs at various concentrations (**Figure 2**). EC50 and TC50 values were estimated based on the curve fitting the concentration-response data. The mean EC50 values of SHFa-2 and SHFb-1 were 0.0363 and 0.0302 mg/L, respectively (**Figure 2A**). The mean TC50 values of SHFa-2 and SHFb-1 were 6.12 and 6.97 mg/L, respectively (**Figure 2B**). When the two sets of EC50 and TC50 values were combined, the resultant average EC50 and TC50 values were 0.0333 and 6.55 mg/L, respectively. The average therapeutic index (TI) value that was calculated as the ratio of average TC50 to EC50 was 197. The data suggest that *E. faecium* 1449 bacteria are more sensitive to Ag-hydrosol treatment than human nasal epithelial cells. Moreover, Ag-hydrosol NPs are highly safe in that the higher the TI value, the safer is the therapeutically effective dose that can be used before it reaches the threshold toxicity level.

### 3.3 Effect of Ag-Hydrosal NPs on *E. faecium* 1449 viability imaged by confocal microscopy

**Figure 3:**
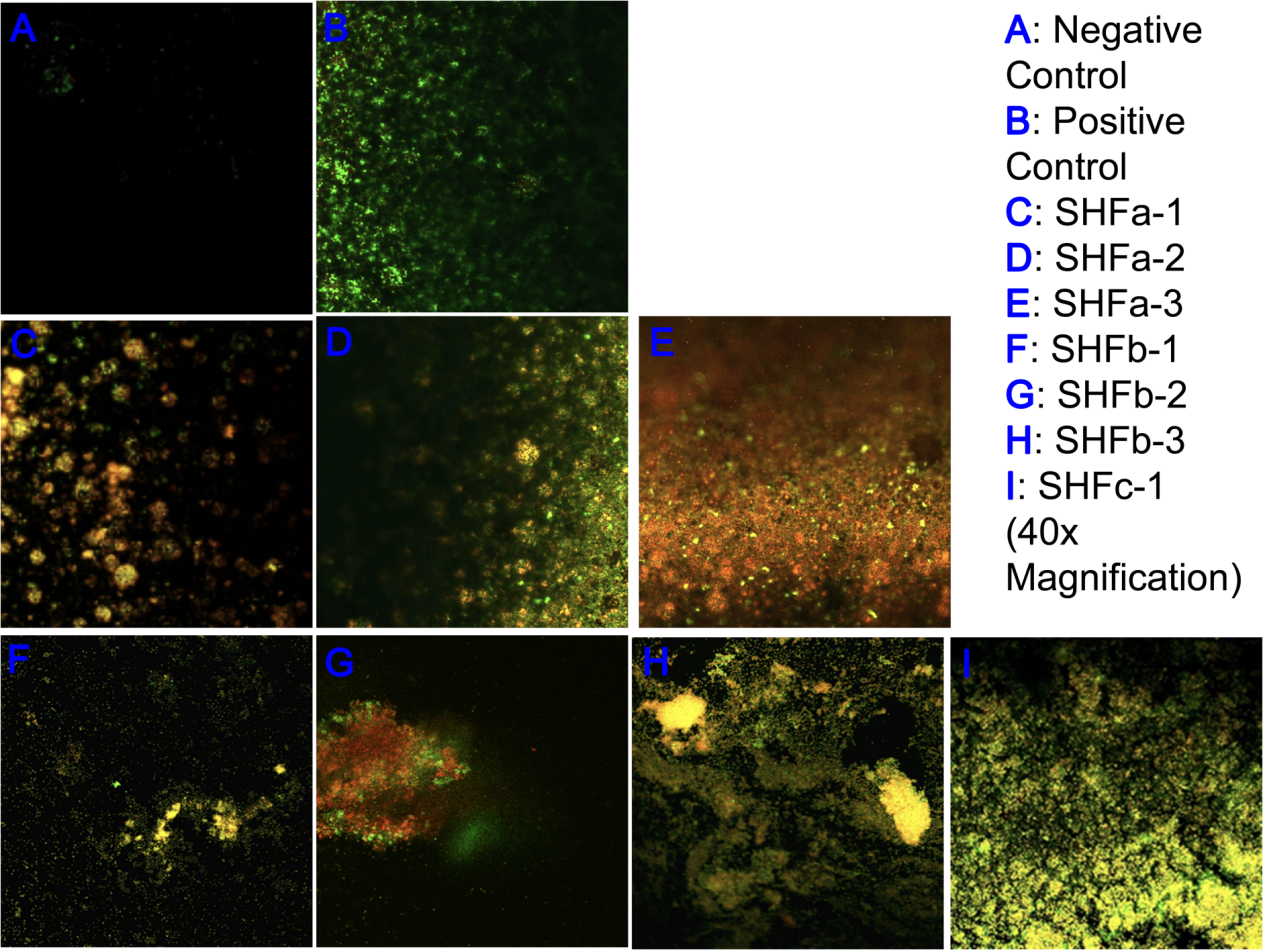
Representative confocal laser scanning microscopy images of **(A)** negative control and **(B)** *E faecium 1449* biofilm matrices treated with vehicle and Ag-hydrosol NP Sample **(C)** SHFa-1 (14.2 mg/L), **(D)** SHFa-2 (14.2 mg/L), **(E)** SHFa-3 (14.2 mg/L), **(F)** SHFb-1 (26.5 mg/L), **(G)** SHFb-2 (26.7 mg/L), **(H)** SHFb-3 (26.8 mg/L) and **(I)** SHFc-1 (123 mg/L). Original magnification 40 ×.

The LIVE/DEAD biofilm viability assay kit (Thermo Fisher Scientific, Waltham, MA) allowed viable and dead bacterial cells to be labeled with the green and red fluorescence signals, respectively. As such, an effective bactericidal treatment should exhibit more red fluorescence signals than green fluorescence signals. As shown in **Figure 3**, the negative control contained no bacterial biofilm and thus had relatively low levels of green and red fluorescence signals (**Figure 2A**). The positive control contained untreated *E. faecium* 1449 biofilm and thus had a relatively more green fluorescence signals and a relatively less red fluorescence signals (**Figure 3B**). Red fluorescence signals that appeared in the positive control images were due to random cell death within the bacterial biofilm. Treatment with SHFa-3 (12.4 mg/L; **Figure 3E**) and SHFb-2 (26.8 mg/L; **Figure 3G**) Ag-hydrosol NPs in *E. faecium* 1449 appeared to have a relatively high bacterial killing efficacy, elucidated by a higher proportion of dead cells indicated by red fluorescence signals than viable cells indicated by green fluorescence signals. Treatment with SHFa-1 (12.4 mg/L; **Figure 3C**), SHFa-2 (12.4 mg/L; **Figure 3D**), SHFb-1 (26.5 mg/L, **Figure 3F**), SHFb-3 (27.0 mg/L; **Figure 3H**) and SHFc-1 (123 mg/L; **Figure 3I**) Ag NPs appeared to have similar amount of green fluorescence signals relative to red signals, suggesting similar bactericidal effectiveness.

### 3.4 Extracellular *polymeric substance* (EPS) formation in *E. faecium* 1449 biofilms imaged by confocal microscopy

**Figure 4:**
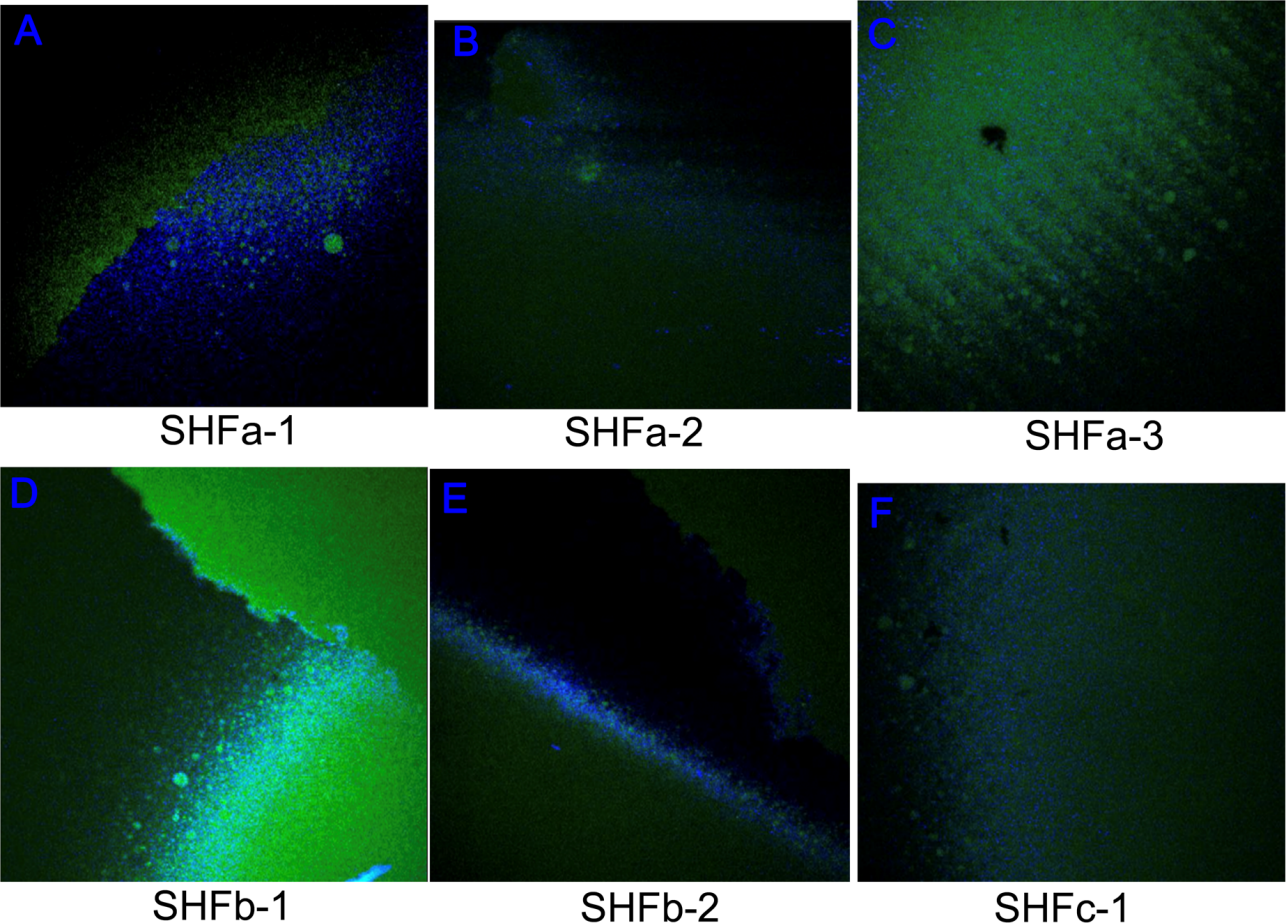
Representative confocal laser scanning microscopy images of extracellular polymeric substances comprising *E faecium 1449* biofilm matrices following the treatment with Ag-hydrosol NP Sample **(A)** SHFa-1, **(B)** SHFa-3, **(C)** SHFa-3, **(D)** SHFb-1, **(E)** SHFb-2 and **(F)** SHFc-1. Original magnification 40 ×.

EPS is an indicator of the level of stress and reaction of the biofilm when exposed to a drug *in vivo* as self-defense mechanism (Yang et al., 2022). As shown in **Figure 4**, treatment with Ag NPs elicited great amounts of polysaccharide (blue fluorescence signals) and protein (green fluorescence signals) that constitute the EPS of *E. faecium* 1449 biofilms. The surface polysaccharide coverages were clearly visible in all Ag-NP treated biofilms (**Figure 4**). Given the fact that EPS serves as the defensive barrier utilized by bacteria to maintain biofilm integrity and prevent biocides from penetrating into the biofilm (Zhou et al., 2015), this result suggests that treatment with Ag NPs exposes the biofilm-forming *E. faecium* 1449 to the bactericidal environment, eliciting the defensive reaction of the bacteria. Overall, the result displays a high surface coverages of EPS, denoting a substantial stressful conditions caused by Ag-hydrosol NPs and its high potency against MDR biofilms.

### 3.5 Spectrofluorimetric quantification of biofilm reduction

As shown in **Table 1**, the *in vitro* bactericidal effect of Ag NPs expressed by percent biofilm reduction in descending order was: SHFb-2 > SHFc-1 > SHFa-3 > SHFb-3 > SHFb-1. Nonetheless, no statistically significant difference in the percent biofilm reduction was found among different Ag-hydrosol NP batches at different concentrations (P = 0.809 using one-way ANOVA). Since the percent biofilm reduction values for all Ag NP concentrations tested were approaching 100%, the EC50 value was not readily to be calculated.

**Table 1:** Results of percent biofilm reduction in initial trials.

Ag NP Sample ID	Ag NP Concentration (mg/L)	% Biofilm Reduction
SHFa-3	12.4	88.12 ± 20.58
SHFb-1	26.5	78.09 ± 23.07
SHFb-2	26.8	95.20 ± 8.32
SHFb-3	27.0	81.06 ± 32.80
SHFc-1	123	94.02 ± 10.36

The data represent the standard deviation (SD) of the mean from 3 independent experiments (N = 3).

### 3.6 TEM interpretation

**Figure 5:**
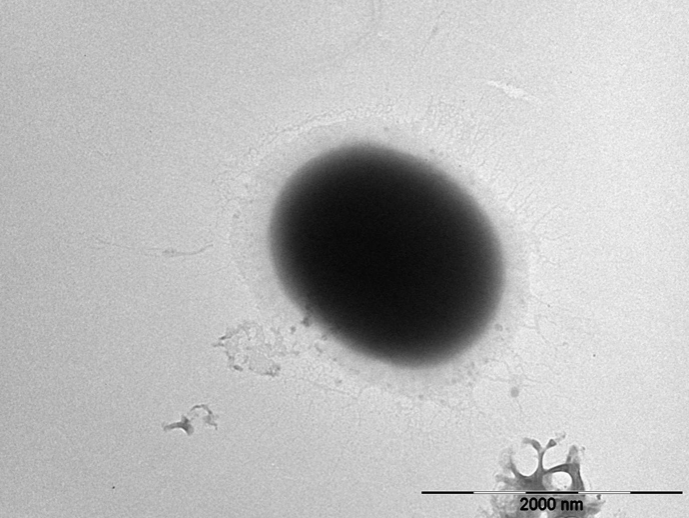
Representative images of **(A)** the untreated *E. faecium 1449* cell (scale bar = 2000 nm).

**Figure 6:**
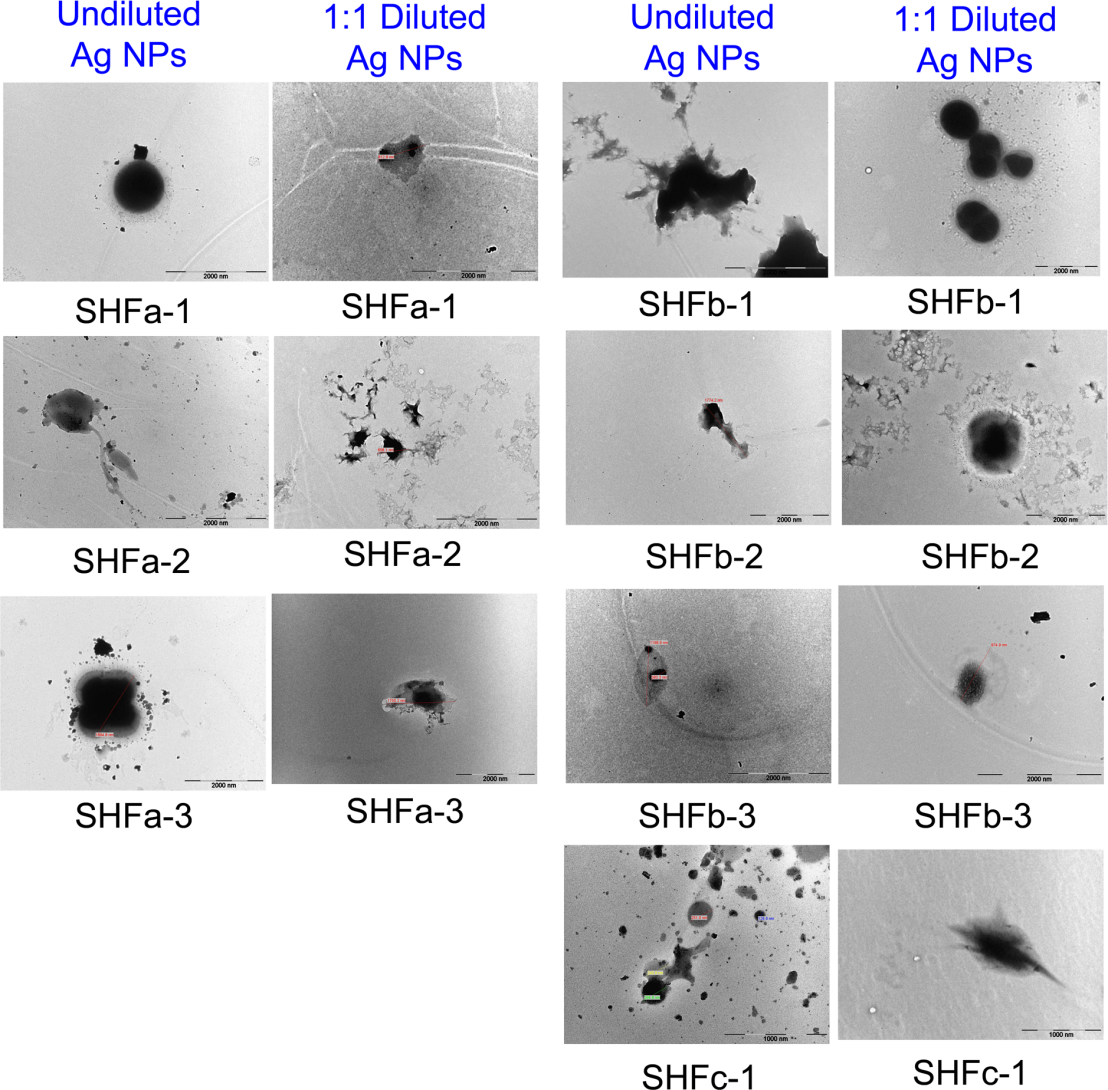
Representative TEM images of *E. faecium 1449* cells treated Ag-hydrosol NPs. Scale bar = 1000 nm for SHCc-1 samples. Scale bar = 2000 nm for all other samples.

The TEM image of *E. faecium* 1449 revealed a coccus-shaped bacterium expressing pili (**Figure 5**). This observation is consistent with our previous report (Limayem et al., 2020). Representative TEM images in **Figure 6** demonstrated the death of *E. faecium* 1449 cells in all treatment samples following the Ag NP treatment at various concentrations for 3 hours. Dead *E. faecium* 1449 bacteria showed graying of the interior of the bacterial cell, which represented a loss of electron density. Moreover, when compared with the untreated *E. faecium* 1449 bacteria (**Figure 5**), the cocci shape of some bacterial cells appeared to be disrupted in the presence of Ag NPs (**Figure 6**), which could be due to the oxidative stress on the bacterial cell membrane or physical membrane damage caused by Ag NPs. Furthermore, TEM images revealed the trapping or wrapping of microbes by Ag NPs, suggesting the accumulation of Ag NPs in EPS surrounding the *E. faecium* 1449 cell.

### 3.7 AI interpretation

**Figure 7:**
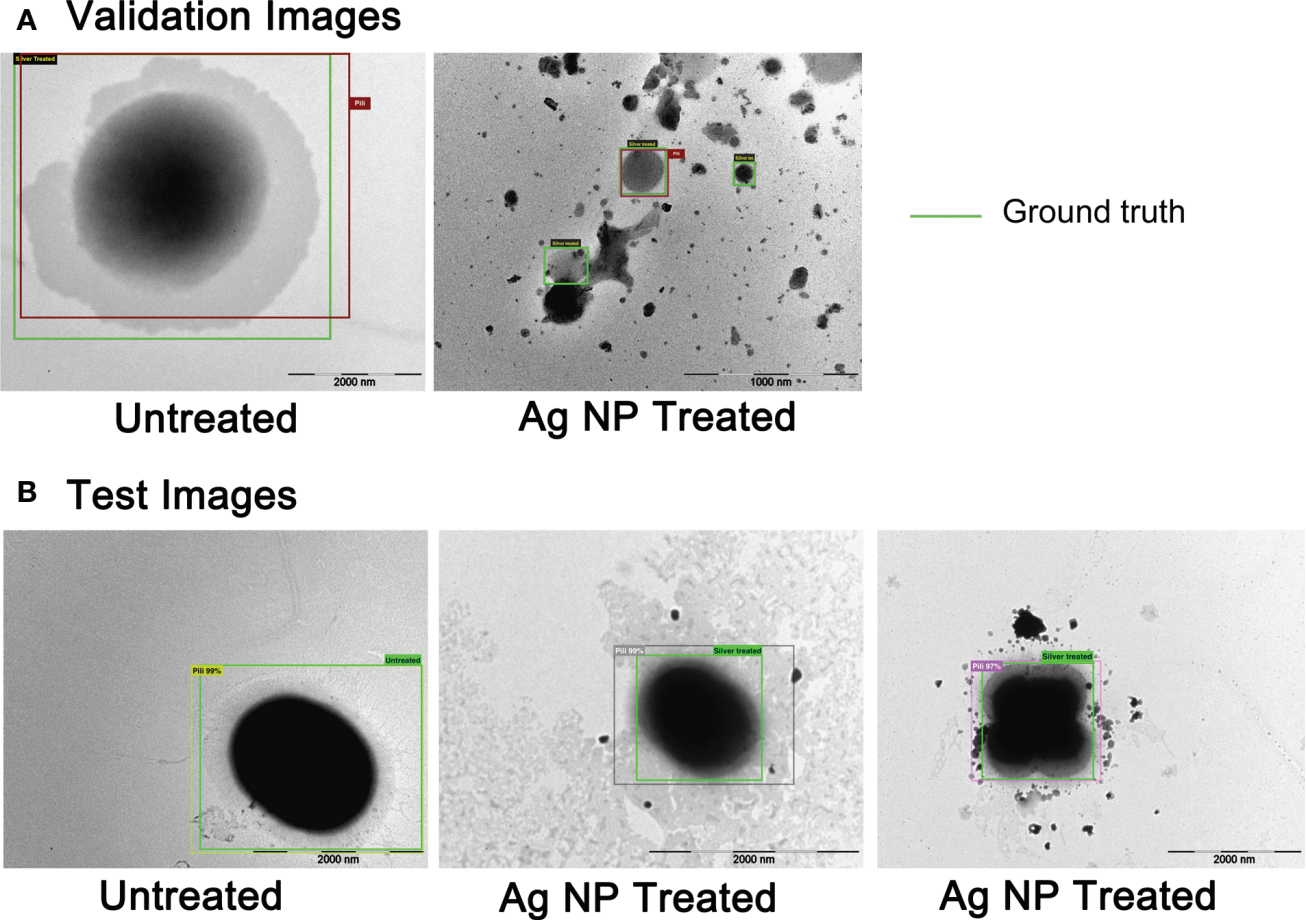
The AI detection of untreated and treated *E faecium 1449* in TEM images based on R-CNNs technique. **(A)** TEM images for validation. **(B)** TEM images for testing. The ground truth boxes were in green color, while boxes automatically identified by AI were in different colors other than green.

Results of *E. faecium 1449* detection in TEM images conducted by the fast R-CNN deep learning method are shown in **Figure 7**. The green boxes were the manually emplaced ones (called ground truth), while the boxes with different colors other than green were the ones automatically identified by our neural network architecture as the foreground (i.e., pili for bacteria). **Figure 7A** shows the results for validation images, while **Figure 7B** shows those for testing images. All of the validation and testing images were unseen by the AI model during training. As shown in **Figure 7B**, the positions of the ground truth boxes were very closely matching the positions of automatically emplaced boxes, indicating the correctness of the technique development for this specific case. The confidence of detection was indicated as a percentage, which was not less than 97% accuracy. The AI approach developed for this study therefore allowed automated detection and immediate observation of microorganisms with normal or altered morphology, revealing high accuracy upon the production of the images that would otherwise take a long time even from trained eyes to observe them with the Ag-hydrosol NPs in a convoluted TEM image.

## 4 Discussion

The development of metallic nanoparticles as the means to overcome antibiotic resistance is currently an area of intense research (Lee et al., 2019; Pathania et al., 2022; Pathania et al., 2022). Like other metal nanoparticles, the size of Ag NPs is a key determinant of their membrane permeability and biological fate (Wu et al., 2019). In this study, the TEM-determined core sizes of Ag-hydrosol NPs were in the 1 ~ 10 nanometer range (**Figure 1**), implicating a high surface-area-to-volume ratio that is likely to contribute to the potent antibacterial activity of Ag-hydrosol NPs associated with cell wall penetration and membrane damage.

Quantitative evaluation of the anti-biofilm activity and cytotoxicity of overnight Ag-hydrosol NPs treatment at various concentrations in *E. faecium* 1449 bacteria and human nasal epithelial cells resulted in a mean EC50 value of 0.0333 mg/L and a mean TC50 value of 6.55 mg/L, respectively (**Figure 2**), and thus the calculated TI value was 197. Since the clinical TI of the Ag-hydrosol NP product is unknown, estimation the TI using *in vitro* assays is of prime priority since it is the major indicator of the likelihood successful development of a treatment. The high TI value of the Ag-hydrosol NP product suggests that it has a promising safety and efficacy profile.

Results of confocal laser scanning microscopy revealed that *E. faecium 1449* biofilms contained a rich portion of EPS irrespective of the concentrations of Ag NPs used (**Figure 3**). This observation was similar to that of our previous study, in which treatment with nanomicelle chitosan-zinc oxide composites elicited a greater amount of polysaccharide and proteins that comprise the EPS matrix of *E. faecium 1449* biofilms than the vehicle control treatment (Limayem et al., 2020). Although the exact mechanism underlying the augmenting effect of silver on biofilm formation is unclear, it is likely that treatment with Ag-hydrosol NPs promotes *E. faecium 1449* biofilm formation as a survival mechanism in response to the antimicrobial stress elicited by the treatment. The EPS matrix of biofilms usually acts as a barrier protecting bacteria against stress from exogenous toxicants, including antibiotics (Stewart and Costerton, 2001; Wang et al., 2018). As such, it is conceivable that the EPS matrix would prevent Ag NPs from penetrating the bacterial cells and antagonize the bactericidal activity of the NPs (Kang et al., 2014). However, the increased EPS did not seem to reduce the antibacterial effect of Ag NPs in this study. Results of both the confocal microscopy and biofilm reduction quantification showed that different batches of Ag-hydrosol NPs at concentrations ranging from 12.4 mg/L to 123.0 mg/L consistently exhibited marked bactericidal activity against the MDR *E. faecium 1449* strain (**Figure 2A**, **Figure 3** and **Table 1**). The mean percent deduction of biofilms of 3D *E. faecium 1449* cultures ranged from 78.09% ~ 95.20% with no significant difference in the mean percent biofilm deduction values being observed among all Ag NP samples (P = 0.809) (**Table 1**). SHFa and SHFb despite having significantly lower Ag concentration, were demonstrated to be as effective as SHFc. The SHFa and SHFb all silver contents as sub-10 nm particles, whereas SHFc has majority of its silver content as 25-50 nm particles (**Figure 1E**). This result agrees with previous studies in which the smaller AgNPs were shown to exhibit high antimicrobial activity (Morones et al., 2005; Bin Ahmad et al., 2012; Raza et al., 2016).

It is postulated that the presence of EPS improves the surface accumulation of Ag NPs although the bacterial cell internalization of Ag NPs may be limited by EPS (Gao et al., 2021), and Ag NPs generate free radicals when coming into contact with the bacteria, and the free radicals make the cell membrane porous, leading to the bacterial cell death (Kim et al., 2007; Lee et al., 2019). Further studies are warranted to elucidate the mechanism by which the bactericidal effect of Ag-hydrosol NPs is enhanced through the interaction between the NPs and EPS matrix.

Under TEM, the distribution of Ag NPs was visible in the surrounding area of *E. faecium 1449* bacterial cells following the 3-hr Ag NP treatment (**Figure 6**). The presence of Ag NP clusters surrounding the bacterial cells can be interpreted as the EPS-improved cell surface accumulation of Ag NPs. Moreover, some *E. faecium 1449* bacterial cells appeared elongated in shape (**Figure 5**), suggesting Ag NP induced degenerative changes to *E. faecium 1449* bacteria.

Unlike the gram positive wild-type *E. faecium*, the MDR *E. faecium 1449* strain was found to express pili in this study (**Figure 5**) as well as in our previous study (Limayem et al., 2020). Pili in Gram-positive *E. faecium* have been implicated in the pathogenesis and resistance of Enterococcus species (Hendrickx et al., 2009; Arias and Murray, 2012). They are important virulence factors with the ability to facilitate microbial attachment to host cells and thus initiate and sustain infection (Arias and Murray, 2012). Based on this feature, an AI approach was developed in this study to detect the *E. faecium 1449* bacterium by identifying features that represent the foreground object of interest, *i.e.*, a coccus-shaped bacterium with pili on the surface (**Figure 7**). The Fast R-CNN method allowed us to achieve the human-expert-level accuracy (97% ~ 99%) and rapid detection of *E. faecium* 1449 bacterium in TEM data. This approach represents an image analysis framework that works on TEM images. It can be potentially used to determine the bactericidal effect of Ag NPs by counting the live and dead bacterial cells within the TEM images when a large amount of cytotoxicity TEM data are available for training. Since the *E. faecium* 1449 bacterium imaged with TEM exemplifies general features of many microbes, the established AI approach can be readily extended to handle different types of bacteria.

## 5 Conclusions

While several studies have demonstrated the effectiveness of silver nanocomponents on bacteria (Gong et al., 2007; Rai et al., 2009; Guzman et al., 2012), little is known about the new bioactive Ag-hydrosol NPs, being a safe and promising nanotherapeutic agent combatting MDR microbial contaminations. In this study, the bactericidal effect and cytotoxicity of the Ag NPs were examined using the confocal laser scanning microscopy, TEM and Biotek Synergy Neo2 multi-mode reader. Our results showed that treatment with the commercially manufactured Ag-hydrosol NPs at concentrations ranging from 12.4 to 123 mg/L resulted in 78.04% ~ 95.20% reduction of *E. faecium 1449* biofilms. The mean TC50 of different batches of Ag NPs determined in human nasal epithelial cells was 6.55 mg/L, which was much higher than the mean EC50 value of 0.0333 mg/L in *E. faecium 1449*, resulting in a TI value of 197. The high TI value indicates a low risk of toxicity within the therapeutic dose range. The approach developed in this study allowed the accurate automated detection of *E. faecium 1449* bacterial cells, setting the stage for an automated detection of a wide range of microbes as well as a rapid evaluation of drug effectiveness.

## Data availability statement

The original contributions presented in the study are included in the article. Further inquiries can be directed to the corresponding authors.

## Author contributions

Conceptualization, AL, SC, and QZ; methodology, AL, MM, NK, DK, FA, NQ, and QZ; software, FA and SC; validation, AL, NQ, and QZ; formal analysis, AL and QZ; investigation, AL, MM, NK, DK, FA, NQ, and QZ; resources, AL, NQ, and QZ; data curation, AL, NQ, and QZ; writing-original draft preparation, AL, SC, NQ, and QZ; writing-review and editing, AL and QZ; supervision, AL, SC, and QZ; project administration, AL and QZ; funding acquisition, AL and QZ. All authors have read and agreed to the published version of the manuscript.
